# The Association Between Intraoperative Compromised Intestinal Integrity and Postoperative Complications in Cancer Patients

**DOI:** 10.1245/s10434-023-14857-7

**Published:** 2024-01-15

**Authors:** Sharon Hendriks, Monique G. Huisman, Suzanne C. Stokmans, Matthijs Plas, Hanneke van der Wal-Huisman, Barbara C. van Munster, Anthony R. Absalom, Gertrude J. Nieuwenhuijs-Moeke, Geertruida H. de Bock, Barbara L. van Leeuwen, Jacco J. de Haan

**Affiliations:** 1 Department of Surgery, University of Groningen, University Medical Center Groningen, Groningen, The Netherlands; 2 Department of Internal Medicine, University of Groningen, University Medical Center Groningen, Groningen, The Netherlands; 3 Department of Anesthesiology, University of Groningen, University Medical Center Groningen, Groningen, The Netherlands; 4 Department of Epidemiology, University of Groningen, University Medical Center Groningen, Groningen, The Netherlands; 5 Department of Medical Oncology, University of Groningen, University Medical Center Groningen, Groningen, The Netherlands

## Abstract

**Background:**

Because of perioperative splanchnic hypoperfusion, the gut wall becomes more permeable for intraluminal microbes to enter the splanchnic circulation, possibly contributing to development of complications. Hypoperfusion-related injured enterocytes release intestinal fatty acid binding protein (I-FABP) into plasma, which is used as proxy of intestinal integrity. This study investigates the occurrence of intestinal integrity loss during oncologic surgery, measured by I-FABP change. Secondary the relationship between compromised intestinal integrity, and related variables and complications were studied.

**Methods:**

Patients undergoing oncologic surgery from prospective cohort studies were included. Urine I-FABP samples were collected preoperatively (T0) and at wound closure (T1), and in a subgroup on Day 1 (D1) and Day 2 (D2) postoperatively. I-FABP dynamics were investigated and logistic regression analyses were performed to study the association between I-FABP levels and patient-related, surgical variables and complications.

**Results:**

A total of 297 patients were included with median age of 70 years. Median I-FABP value increased from 80.0 pg/mL at T0 (interquartile range [IQR] 38.0–142.0) to 115 pg/mL at T1 (IQR 48.0–198.0) (*p* < 0.05). Age (odds ratio [OR] 1.05, 95% confidence interval [CI] 1.02–1.08) and anesthesia time (OR 1.13, 95% CI 1.02–1.25) were related to stronger I-FABP increase. When comparing I-FABP change in patients experiencing any complications versus no complications, relative I-FABP change at T1 was 145% of T0 (IQR 86–260) versus 113% (IQR 44–184) respectively (*p* < 0.05).

**Conclusions:**

A significant change in I-FABP levels was seen perioperatively indicating compromised intestinal integrity. Age and anesthesia time were related to higher I-FABP increase. In patients experiencing postoperative complications, a higher I-FABP increase was found.

**Supplementary Information:**

The online version contains supplementary material available at 10.1245/s10434-023-14857-7.

Surgery, sole or combined with other treatments, is currently the most important curative treatment for solid tumors.^1,2^ Several risk factors are associated with adverse outcomes after oncologic surgery. During the past decades, perioperative loss of intestinal integrity has been considered to be an important risk factor in the development of postoperative complications like sepsis.^3^ Clinical observations show that the gut wall becomes more permeable during and after surgery. Microbes causing sepsis after surgery are often identical to microbes cultured from the mesenteric lymph nodes.^4,5^ Therefore, it was postulated that barriers that prevent intraluminal microbes from entering the splanchnic circulation become compromised during major surgery or following severe trauma.^4,5^ Splanchnic hypoperfusion is an important contributor to the loss of intestinal integrity during surgery and trauma.^6,7^ Intraoperative splanchnic hypoperfusion may be a consequence of hypotension as a result of vasodilating properties of anesthetic drugs or a profound inflammatory response or acute blood loss.^8^ Enterocyte injury and increased gut wall permeability were shown to develop early after hypoperfusion of the splanchnic area resulting in diminished intestinal integrity.^6^

It is possible to measure enterocyte injury by measuring intestinal fatty acid binding protein (I-FABP) in plasma and urine. I-FABP is a small cytosolic protein (14–15 kDa) expressed by mature enterocytes that are found at the tips of the villi of the intestine. Following enterocyte injury, these proteins are readily measurable in plasma and urine.^9–11^ I-FABP levels have been reported to correlate with histological status of the epithelium after intestinal ischemia-reperfusion.^12,13^ Enterocyte injury and changes in intestinal integrity during surgery for solid malignancies have not been described yet.

Therefore, the primary objective of this exploratory study was to investigate the occurrence of intestinal integrity loss during surgery for solid tumors. Secondary objectives were to study the association between patient- and surgery-related factors, including peroperatively measured blood pressure and intestinal integrity, as well as the relationship between intestinal integrity levels and the occurrence of postoperative complications.

## Methods

### Ethics Statement

The data for this study were obtained from two prospective cohort studies: “PICNIC” (PostoperatIve Cognitive dysfunctioN In elderly Cancer patients), and “PICNIC B-HAPPY” (Biomarkers and HAndgrip strength as Predictors for Postoperative outcome in PICNIC) conducted from July 2010 until April 2014 and from August 2014 until March 2017 respectively at the University Medical Center Groningen (UMCG). These studies are registered in the Dutch Clinical Trial database at www.trialregister.nl: NL4219 (2010-07-22) and NL4441 (2014-06-01). Approval was obtained from the local ethics committee. Patient characteristics from both cohorts were described previously.^14–17^ Data collection was conducted according to the revised version of the Declaration of Helsinki (October 2013, Brazil).

### Study Design and Participants

Patients admitted to the UMCG for an elective resection of a solid tumor in two prospective, cohort studies were recruited. In the PICNIC study, only adults aged 65 years and older were included. In PICNIC B-HAPPY, adults aged 18 years and older were included. For the current study, patients were excluded if urine I-FABP samples were incomplete. Based on the fact that I-FABP is mainly produced in the small intestine, resection of part of the small intestine could possibly influence I-FABP changes more than other types of surgery. Therefore, patients undergoing surgery off the small intestine were excluded.^10^ This includes for example Whipple procedures and right hemicolectomy.

### Urine Sampling and I-FABP Measurements

Urine samples were collected before induction of anesthesia (T0) and at wound closure (T1). In addition, in the PICNIC B-HAPPY cohort urine samples were collected on the first day (D1) and second day (D2) postoperatively. Samples were collected directly from a urinary catheter in situ, to ensure a fresh sample. Collected samples were centrifuged for 5 min on 1300xG and cell-free supernatants were stored at −80 °C within 1 hr of urine collection until analysis. Urine I-FABP measurements were performed batchwise by Haemoscan (Groningen, the Netherlands) using an Enzyme-Linked Immuno Sorbent Assay (ELISA) according to the manufacturer’s instructions (HyCult Biotech, Uden, the Netherlands). I-FABP concentration in urine is presented as pg/mL. The detection level of I-FABP is 47 pg/mL. No validated cutoff levels are known.

### Definitions and Data Collection

Patient characteristics, including biological sex, age, body mass index (BMI), comorbidities, tumor site, disease stage, surgical characteristics, and perioperative clinical data, were prospectively collected from medical records. Race and ethnicity data were not collected in this study. In a subgroup, arterial blood pressure (ABP) was recorded every 30 s during surgery. To determine the duration of intraoperative hypotension, a cutoff point for MAP was selected to calculate the time under threshold (TUT) in min. Prolonged exposure for MAP < 65 mmHg was associated with elevated risks of any end-organ injury in noncardiac surgery.^8,18^ Therefore, the TUT was calculated for total time of MAP <65 mmHg. Comorbidities were assessed by using the Charlson comorbidity index.^19^ Type of surgery was dichotomized into intra- and extracavitary surgery, procedure duration was analyzed per hour. Use of any vasoactive medication (noradrenaline, adrenaline, fenylephidrine) during surgery was dichotomized as yes or no. Postoperative complications occurring within 30 days after surgery were scored according to the Clavien-Dindo classification.^20^ Major complications were defined as Clavien-Dindo score 3 or higher. Postoperative complications also were retrospectively categorized as either inflammatory or noninflammatory origin. Complications in which the immune system was involved were considered inflammatory complications. These were defined as infectious complications when diagnostic tests confirmed the occurrence of an infection (by cultures, radiographic findings, or laboratory testing) or when therapy was initiated by clinical suspicion of an infection. If complications were considered as inflammatory, but noninfectious, such as postoperative ileus, delirium, and anastomotic leakage, they were assigned to the inflammatory–noninfectious subgroup.^16,21–23^ Patients were assigned to the inflammatory complications group if at least one of the complications was inflammatory.

Urine I-FABP levels were analyzed as absolute levels and as percentage of T0. Because no cutoff levels are known to distinct a strong increase or minimal to moderate increase, the median percentage increase in I-FABP levels in the current study was defined as the cutoff point to divide patients in two groups. When an I-FABP value was below the detection limit in the first measurement, no percentage could be calculated and, in this case, patients were assigned to the mild to moderate increase or high increase group based on an absolute increase in urine I-FABP levels relative to the median increase in urine I-FABP levels.

### Statistical Analysis

Categorical data were reported as absolute numbers and percentages. For continuous data, mean and standard deviation (SD) are shown when data are normally distributed, and median and interquartile range (IQR) when data are not normally distributed. Differences in patient and surgical variables were tested using independent samples *t*-test, Mann-Whitney *U* test, Chi-square test, or Fisher’s exact test where appropriate. Wilcoxon signed-rank test was used to compare urine I-FABP levels postoperatively to levels preoperatively. A univariate logistic regression was performed to define which variables were associated with a urine I-FABP change, as there are no known predictors.^24^ Variables that were associated with urine I-FABP change in univariate analysis (*p* < 0.10) were taken into multivariable logistic regression analysis. No backwards/forwards selection was used as this study was designed to discover variables associated with I-FABP change, not to build a model predicting I-FABP change*.* The same regression analyses were performed in the subgroup with MAP measurements and in a subgroup of only patients undergoing intracavitary surgery. To compare the change in I-FABP levels in patients who developed complications (all complications, inflammatory, and/or infectious complications) and in patients who did not, a Mann-Whitney *U* test was performed. Given the wide range of tumors and types of surgery, the number of patients with specific complications per type of tumor and surgery was too limited to allow for analysis.^25^

## Results

### Patient Characteristics

**Fig. 1:**
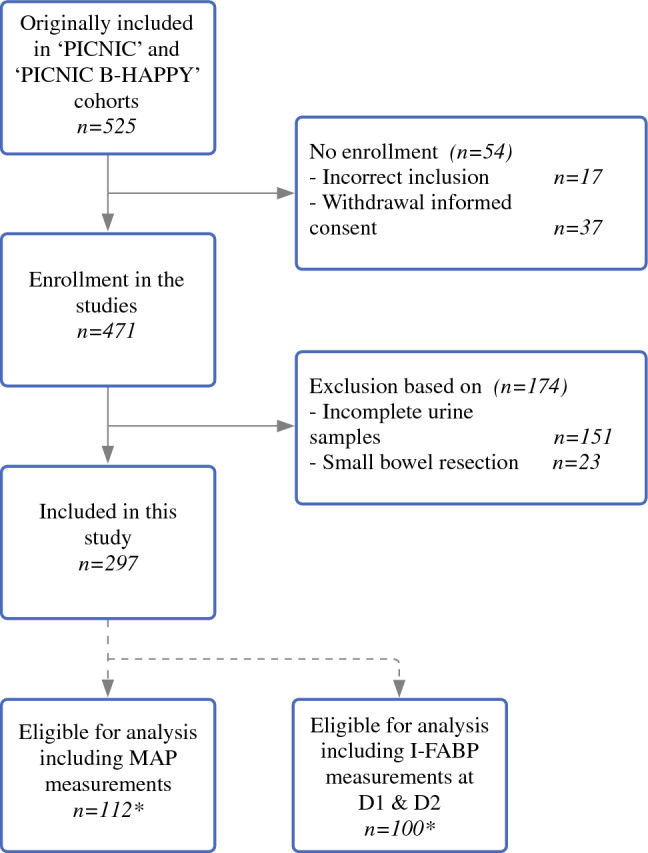
Patients and subgroups. *There is an overlap in patients between subgroups

Of the 525 patients originally included in the PICNIC & PICNIC B-HAPPY cohorts, 297 patients were eligible for this study (Fig. 1). Main reasons for exclusion were incomplete urine I-FABP samples (*n* = 151) patients and small-bowel resections (*n* = 23). A subgroup of 112 patients was eligible for analysis of MAP measurements. In 100 patients, urine I-FABP analysis was performed at D1 and D2 postoperatively. A subgroup of 212 patients underwent intracavitary surgery.

Median age was 70 years (IQR 66–76), and 143 (47.1%) of the patients were female (Supplementary Table 1). Median CCI score—malignancies not included—was 1 (IQR 0-2). In 108 (36.5%) patients, the medical history included peripheral vascular disease.

The median age and CCI scores were similar in patients with and without MAP measurements (*p* = 0.85 and 0.08 respectively). The subgroup of patients with MAP measurements consisted of more women and more patients with a history of peripheral vascular disease compared with the group of patients without MAP measurements (*p* < 0.05). In the subgroup of only patients that underwent intracavitary surgery, tumor type varied. Most patients who underwent intracavitary surgery had gastrointestinal tumors, whereas most patients who underwent superficial surgery had skin, soft tissue, or lymph node tumors (*p* < 0.05).

### Intestinal Integrity During Surgery and Factors Associated

**Fig. 2:**
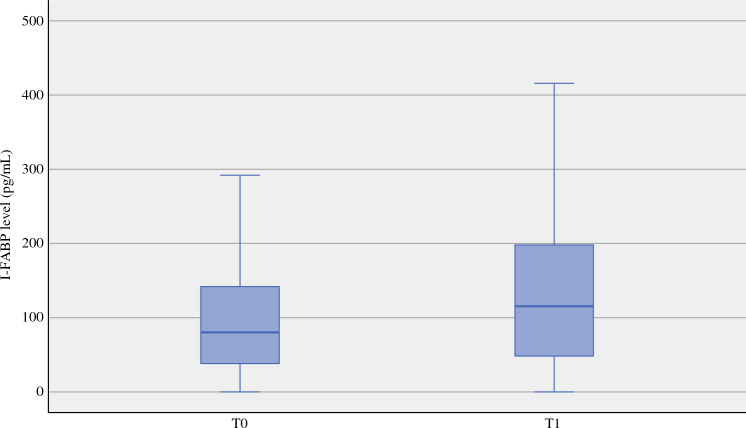
Urine I-FABP levels per time point in pg/ml (*n* = 297). The difference is significant between the moments of measuring

At T0, median I-FABP level was 80.0 pg/mL (IQR 38.0–142.0) and increased to 115.0 pg/mL (IQR 48.0–198.0) at T1 (*p* < 0.05) (Fig. 2). Median I-FABP level at T1 as percentage of the level at T0 was 124.8% (IQR 67.7–235.6). The low I-FABP increase group was defined as an increase of <124% compared with T0 levels, whereas the strong/high I-FABP increase group was defined as T1 I-FABP levels ≥ 124% of T0 levels. In total, 147 (49.5%) patients were in the low I-FABP increase group. In patients with ABP measurement, 57 (50.9%) were in the high I-FABP increase group.

In the high I-FABP increase, 112 (76.2%) patients underwent intracavitary surgery compared with 94 (67.2%) patients in the low increase group (*p* < 0.05). Procedure duration was longer in the high I-FABP increase group with a high I-FABP increase compared with the low increase group (229 min (IQR 124–418) vs. 176 min (IQR 125–305, *P* < 0.05; Supplementary Table 2). In the subgroup of patients with MAP measurements, no difference was found when comparing the TUT of MAP (Supplementary Table 2).

In univariate logistic-regression analysis, older age was associated with a high I-FABP increase (OR 1.04, 95% CI 1.01–1.07), as well as intracavitary surgery (OR 1.71, 95% CI 1.03–2.85) and longer procedure duration per hour (OR 1.13, 95% CI 1.03–1.24). In multivariable logistic regression analysis, age (OR 1.05, 95% CI 1.02–1.08) and procedure duration (OR 1.13, 95% CI 1.02–1.25) remained significantly associated with a strong I-FABP increase. In patients where MAP was measured, no factors were individually associated with a high I-FABP increase. In patients who underwent intracavitary surgery, relative increases above 134% were considered as a high I-FABP increase. Variables that were associated with a high I-FABP at T1 in univariate analysis were age (OR 1.04, 95% CI 1.02–1.07, *p* < 0.05), anesthesia time (OR 1.19, 95% CI 0.99–1.43) and any vasopressin given versus none (OR 2.77, 95% CI 0.84–9.13). In multivariate analysis, only age remained significantly associated with high I-FABP increase (OR 1.05, 95% CI 1.01–1.08, *p* < 0.05).

### Intestinal Integrity First- and Second-day Postoperatively

In 100 patients, urine I-FABP levels were measured at D1 and D2. Patients in this subgroup were younger compared with the rest of the patients as these patients derived from the PICNIC BHAPPY cohort (Supplementary Table 1). In this group, median I-FABP level at T0 was 102.5 pg/mL (IQR 70.0–200.3) and I-FABP level at T1 was 92.5 pg/mL (IQR 0.0–181.3) (*p* = 0.05). Median I-FABP level at D1 was 205.0 pg/mL (IQR 82.0–984.8); median I-FABP level at D2 was 373.0 pg/mL (IQR 65.0–2465.8). The median I-FABP levels as percentage of T0 were at D1 144.0% (IQR 95.9–417.3) and at D2 278.3% (IQR 99.8–1103.3).

Patients with a high urine I-FABP level increase at D2 (> 278.3%) were compared with patients with a low increase (< 278.3%). Procedure duration differed significantly between groups with a low and high I-FABP increase at D2, respectively: 176 minutes (IQR 120–345) and 280 minutes (IQR 182–473). The total of transfused fluids, type of surgery, and use of vasoconstrictors did not differ between groups.

In univariate logistic regression analysis, intracavitary surgery (OR 3.63, 95% CI 1.21–6.31) and procedure duration (OR 1.00, 95% CI 1.00–1.01) were associated with a high D2 I-FABP increase. In multivariable regression analysis, none of these variables were associated with a high I-FABP increase at D2. In patients who underwent intra-cavitary surgery, no variables were significantly associated with high I-FABP increase in univariate analyses.

### Intestinal Integrity and Postoperative Complications

**Fig. 3:**
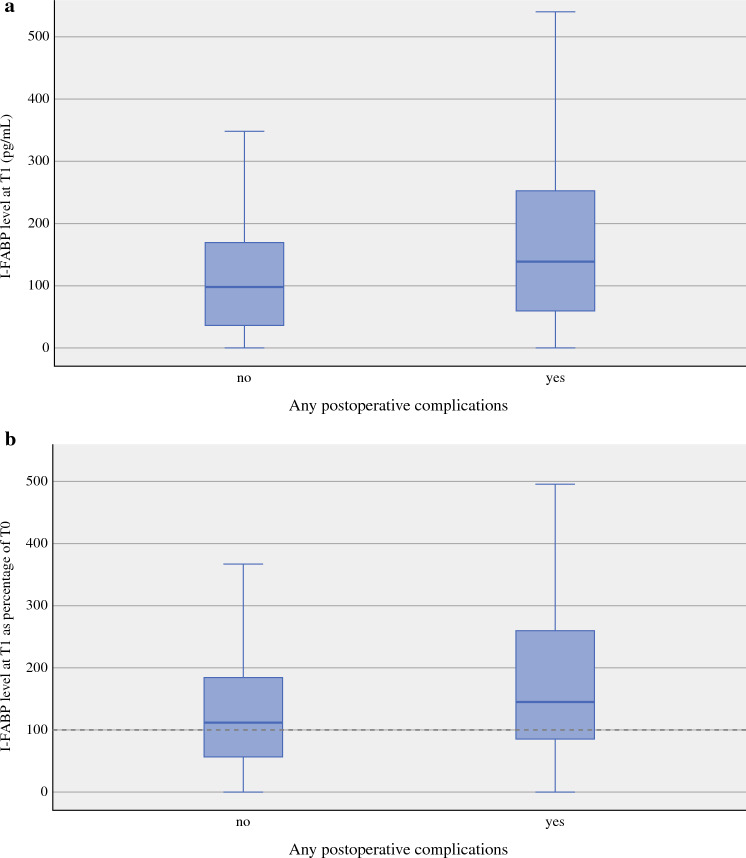
Urine I-FABP levels by patients experiencing any postoperative complications and patients without any postoperative complications. **a** Urine I-FABP levels (pg/mL) at T1 for patients experiencing any postoperative complications and patients who did not. **b** Urine I-FABP levels at T1 as % of value at T0 for patients experiencing any postoperative complications and patients who did not

Postoperative complications occurred in 148 of 297 (49.8%) patients. Most complications were delirium (*n* = 34), wound infections or abscesses (*n* = 28), pneumonia (*n* = 27), and nutrition-related complications, such as gastroparesis (*n* = 25). Major complications occurred in 35 (11.8%) patients. When comparing patients experiencing no complications versus any complications, T1 I-FABP levels were significantly different (98.0 pg/mL, IQR 37.0–169.0; 139 pg/mL, IQR 59.5–252.5 vs. *p* < 0.05) (Fig. 3A). At T0, I-FABP levels did not differ between groups. Median T1 I-FABP levels as percentage of T0 levels were 113% (IQR 55–184) and 145% (IQR 86–260) and for patients experiencing no complication and any complication (Fig. 3B). I-FABP levels at T0, T1, both absolute and relative compared with T0, did not differ for patients experiencing minor or no complications versus patients experiencing major complications.

In total, 62 patients experienced inflammatory–infectious complications (20.9%), 43 patients experienced inflammatory–noninfectious complications (14.5%). I-FABP levels at T1 for patients experiencing inflammatory–infectious complications did not significantly differ from patients without these complications, nor did I-FABP levels at T0. I-FABP levels at T1 differed in the group experiencing inflammatory–noninfectious complications (1444.0 pg/ml, IQR 288.0–3243.0) compared with the group without these complications (222.0 pg/ml, IQR 82.0-898.0; *p* < 0.05) (Supplementary Fig. 1). In patients experiencing inflammatory–infectious complications as well as in patients experiencing inflammatory–noninfectious complications, I-FABP levels at T1 as percentage of levels at T0 (respectively 737%, IQR 113–5975 and 1445%, IQR 306–4793) significantly differed from patients without these complications (respectively 287%, IQR 61–2164 and 302%, IQR 83–3183).

When investigating inflammatory complications in the subgroup of patients who underwent intracavitary surgery, patients experiencing infectious–inflammatory complications had significantly higher relative T1 I-FABP levels compared with T0; 126.7% (80.2–244.0) for patients who did not experience these complications versus 200% (100–324.0) for patients experiencing infectious–inflammatory complications (*p* < 0.05). Also in patients experiencing noninectious–inflammatory complications this was the case with 127.9% (80.5–239.9) versus 227.6% (94.4-409.7) for patients who did not and who did experience noninfectious–inflammatory complications respectivley (*p* < 0.05).

Median I-FABP levels pre- and postoperatively did not significantly differ for patients who died in the first year after surgery compared with those who survived. Also, absolute and relative change of I-FABP peroperatively were not associated with mortality the first year after surgery.

### Complications and Intestinal Integrity Day 1 and 2 Postoperatively

**Fig. 4:**
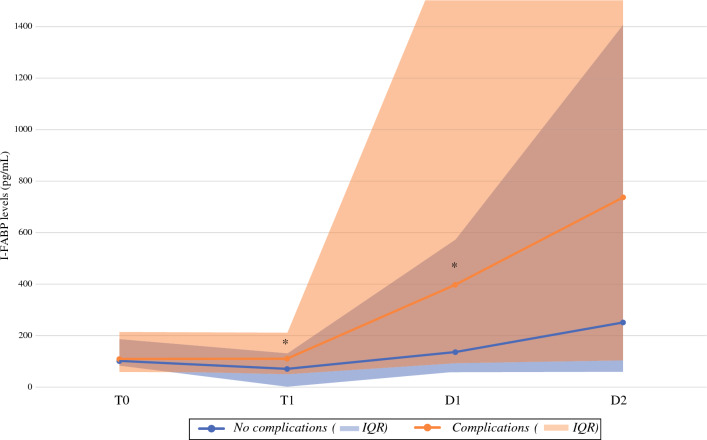
Median I-FABP levels per moment of measurement (n = 100) by groups of patients without 30-day complications and patients experiencing complications. *At moment of measurement significant difference between groups

In the group with measurements at D1 and D2, I-FABP measurements in the group of patients not experiencing and experiencing postoperative complications differed on T1 and D1 with median I-FABP levels of 71.5 pg/mL (IQR 0.0–132.0) and 110.5 pg/mL (48.0–212.0; *p* < 0.05). At D1, urine I-FABP levels were 135.5 pg/mL (63.0–573.0) and 398.5 pg/mL (87.0–1785.0) respectively (*p* < 0.05; Fig. 4). I-FABP measurements in patients experiencing inflammatory–infectious complications significantly differed at T1 (147.0 pg/mL, IQR 77.0–295.0) compared with patients without these complications (79.5 pg/mL, IQR 0.0–154.0) (*p* < 0.05). Levels at T0, D1, and D2, as well as I-FABP levels at T1 and D2 as percentage of T0 did not significantly differ between these groups. For the occurrence of major complications, I-FABP levels at T0, T1, D1, and D2, both absolute and relative levels at T1 and D2 compared with T0, did not significantly differ. For inflammatory–noninfectious complications, I-FABP levels at D1 in patients experiencing these complications (1444.0 pg/mL, IQR 288.0–3242.0) significantly differed from patients without these complications (158 pg/mL, IQR 73.0–630.0) (*p* < 0.05). Levels at T0, T1, D2, and I-FABP levels at T1 and D2 as percentage of levels at T0 did not significantly differ in this subgroup for inflammatory–noninfectious complications versus no inflammatory–noninfectious complications.

## Discussion

This study demonstrates a significant increase in urine I-FABP levels during oncologic surgery indicating a diminished intestinal integrity. This is the first study in oncologic surgery demonstrating this. Increased age and a longer procedure were associated with an increase in postoperative I-FABP levels. A more substantial, postoperative I-FABP increase was found in patients experiencing postoperative 30-day complications compared to patients without complications.

One of the main findings in this study, i.e., compromised intestinal integrity, is an interesting aspect for a more profound understanding of the pathophysiology involved in (oncologic) surgery and the development of complications and a potential direction for future research. A compromised intestinal integrity was associated with older age and longer procedure duration but not with longer TUT of MAP, indicating that hypotension not necessarily is accompanied with splanchnic hypoperfusion. In preceding studies, splanchnic hypoperfusion was found to be a vital contributor to the loss of intestinal integrity during surgery and trauma.^6,7^ In patients with septic shock or critical illness, intestinal mucosal injury possibly derived from gastrointestinal perfusion abnormalities was thought to be a major risk factor for organ failure and poor outcomes.^26–28^

In patients presenting at the emergency department after multiple trauma injuries with a MAP less than 70 mmHg, significantly higher plasma I-FABP concentrations were demonstrated in comparison with patients with a normal (70–99 mmHg) or high (>100 mmHg) MAP or healthy controls.^29^ These studies show an association between splanchnic hypoperfusion and intestinal deterioration and dysfunction. Major difference between these studies and ours is probably the mechanism of hypotension and the body’s response to it. Hypotension due to blood loss in case of trauma is associated with relative hypovolemia with as a consequence splanchnic vasoconstriction and hypoperfusion. In patients with septic shock, the use of high dosages of vasoactive medication is a known cause of splanchnic vasoconstriction and hypoperfusion. In our surgical population, hypotension is frequently the result of vasodilating properties of anesthetics used. The intravascular volume however is tried to maintain at an adequate level with the goal to prevent hypoperfusion of organs. Another key distinction between our study and the above-mentioned studies is that the patients in the earlier studies were younger. A younger age could have influenced the results, as we observed an association between age and strong I-FABP increase during surgery. Age also was the only variable associated with a high I-FABP increase in multivariate regression in patients who underwent intracavitary surgery only. Comparable results were found in patients with acute decompensated heart failure with a mean age of 55.8 years, in whom no significant correlations were found between serum I-FABP levels and invasively measured hemodynamic parameters, such as diastolic and systolic blood pressure.^30^

As people age, there is an increase in the prevalence of vascular illnesses, such as atherosclerosis playing a role in nonocclusive mesenteric ischemia and chronic mesenteric ischemia.^31,32^ Nonacute mesenteric ischemia is not always diagnosed, but intestinal perfusion during surgery might be diminished in these patients and, therefore, potentially affect intestinal integrity. Under these conditions, intestinal perfusion could be compromised due to a higher individual MAP threshold and hypoperfusion could occur even at MAPs that are considered normal. This mechanism could explain why we did not find a relation between strong I-FABP levels postoperatively and TUT for MAP during surgery. In future studies, using an individual threshold MAP for the definition of hypotension based on the patients’ normal blood pressure would be interesting. However, the intraoperative MAP is influenced by factors that also can affect I-FABP levels. The amount and type of fluid given could influence I-FABP concentrations by dilution, and use of vasoactive medication can lead to splanchnic hypoperfusion. No association was found between high I-FABP increase, vasoactive medication administered, and fluids given. However, this should be considered in future studies.

In 148 of 297 (49.8%) patients, postoperative complications occurred. Total complication rates are comparable to other cohorts studying older adults undergoing surgery for solid malignancies.^33,34^ In patients experiencing any 30-day complications postoperatively, I-FABP levels at T1 were significantly higher than in patients without complications. In the subgroup with I-FABP-measurements at D1 and D2, levels at T1 and D1 were significantly lower for patients without 30-day complications compared with patients experiencing 30-day complications, suggesting a co-relation between I-FABP increase and the occurrence of postoperative complications. In patients undergoing open thoracic or thoracoabdominal aortic repair, I-FABP levels were high during surgery. They remained high on the first postoperative day in patients who ultimately developed intestinal necrosis.^35^ In 31 patients undergoing elective major abdominal surgery, an increased plasma I-FABP concentration was found in patients with intestinal cancer compared with patients without intestinal cancer. Further elevation of I-FABP concentrations was found in patients with sepsis.^36^ The relationship between intestinal integrity, I-FABP increase, and postoperative complications is complex and remains unclear. Data from our study and existing literature suggest that measuring I-FABP at a longer than directly postoperatively may be more indicative of the likelihood of postoperative complications.

As perioperative loss of intestinal integrity is considered as an essential risk factor in the development of in particular inflammatory complications, a distinction between noninflammatory, (non-)infectious complications was made.^3–5^ I-FABP levels at T1 were significantly higher for patients experiencing inflammatory–noninfectious complications compared with patients without these complications. Relative T1 I-FABP levels compared with T0 were substantially higher for both inflammatory–infectious complications and inflammatory–noninfectious complications than for patients without these complications. This also was the case in patients who only underwent intracavitary surgery. The results of this study indicate that patients with higher I-FABP levels postoperatively (absolutely and relatively) might be more vulnerable to inflammatory complications. I-FABP serum levels at admission at the ICU (postoperatively) also were predictive of infectious complications in postoperative cardiac surgery patients.^37^

This study has several limitations that need to be addressed. First, there could be a selection bias as more patients in better physical conditions may have participated in this study.^38^ Second, the cutoff points for elevated I-FABP urine levels need to be clarified. In this explorative study, we chose to use median I-FABP levels as cutoff points to have equal groups to compare and get an insight into factors influencing I-FABP levels postoperatively. From studies using plasma I-FABP and urine I-FABP, no clear preference for urine or plasma I-FABP and no precise cutoff levels emerge, making studies difficult to compare. Another limitation is the lack of information on intestinal vascular disease influencing splanchnic blood flow during surgery, which might influence I-FABP levels. Because CT scans before surgery were not performed in the arterial contrast phase, no reliable information could be obtained about preexisting vascular intestinal disease. Furthermore, arterial blood pressure measurements and urine I-FABP levels at D1 and D2 were only available for a subgroup. A strength of our study is the size of this prospective cohort study. A large group of patients was included, and many variables possibly associated with intestinal integrity were studied. To our knowledge, this is the first cohort study to investigate perioperative compromised intestinal integrity in cancer patients.

In this explorative study, our indicate loss of intestinal integrity in oncologic surgery. Future studies should focus on further standardization of I-FABP measurement for better comparison between studies by, for example, measurements on fixed moments and comparing plasma and urine measurements in patients to healthy controls. Before surgery or other interventions, it is important to assess the patients’ intestinal vascular status by screening for abdominal symptoms that may be caused by (chronic) intestinal ischemia and utilizing CT scans with contrast in the arterial phase. In addition, other measurements of intestinal perfusion, for example, gastric mucosal PiCO2 levels, may help to gain more information about the clinical relevance of I-FABP as a marker for intestinal hypoperfusion.

## Conclusions

In patients undergoing surgery for solid malignancies, urinary I-FABP levels at time of skin closure and at D1 and D2 are increased compared to preoperative levels, indicating a compromised intestinal integrity during surgery. A high I-FABP increase was associated with older age and prolonged procedure duration. Interestingly, no association was found between I-FABP increase and intraoperative time of MAP <65 mmHg. In patients experiencing postoperative complications, the I-FABP increase at T1 was significantly higher. However, the relationship between I-FABP levels as proxy of intestinal integrity, factors associated with perioperative diminished intestinal integrity and the risk for developing postoperative complications is complex and largely unclear yet. Future studies with concomitant measurement of mesenteric perfusion and intestinal integrity-related complications should be undertaken to obtain a better insight into the relationship between surgical factors, mesenteric vascular status, intestinal integrity, and postoperative complications in oncologic surgery.

### Supplementary Information

Below is the link to the electronic supplementary material.

Supplementary file1 (DOCX 211 kb)

Supplementary file2 (DOCX 26 kb)
