# Supplementary material for: The Association Between Intraoperative Compromised Intestinal Integrity and Postoperative Complications in Cancer Patients
Source: Ann Surg Oncol. 2024 Jan 15;31(4):2699–708. doi: 10.1245/s10434-023-14857-7 (PMC10908644; doi:10.1245/s10434-023-14857-7)
Supplement: Supplementary file 1 — Supplementary file1 (DOCX 211 kb) [file 10434_2023_14857_MOESM1_ESM.docx]

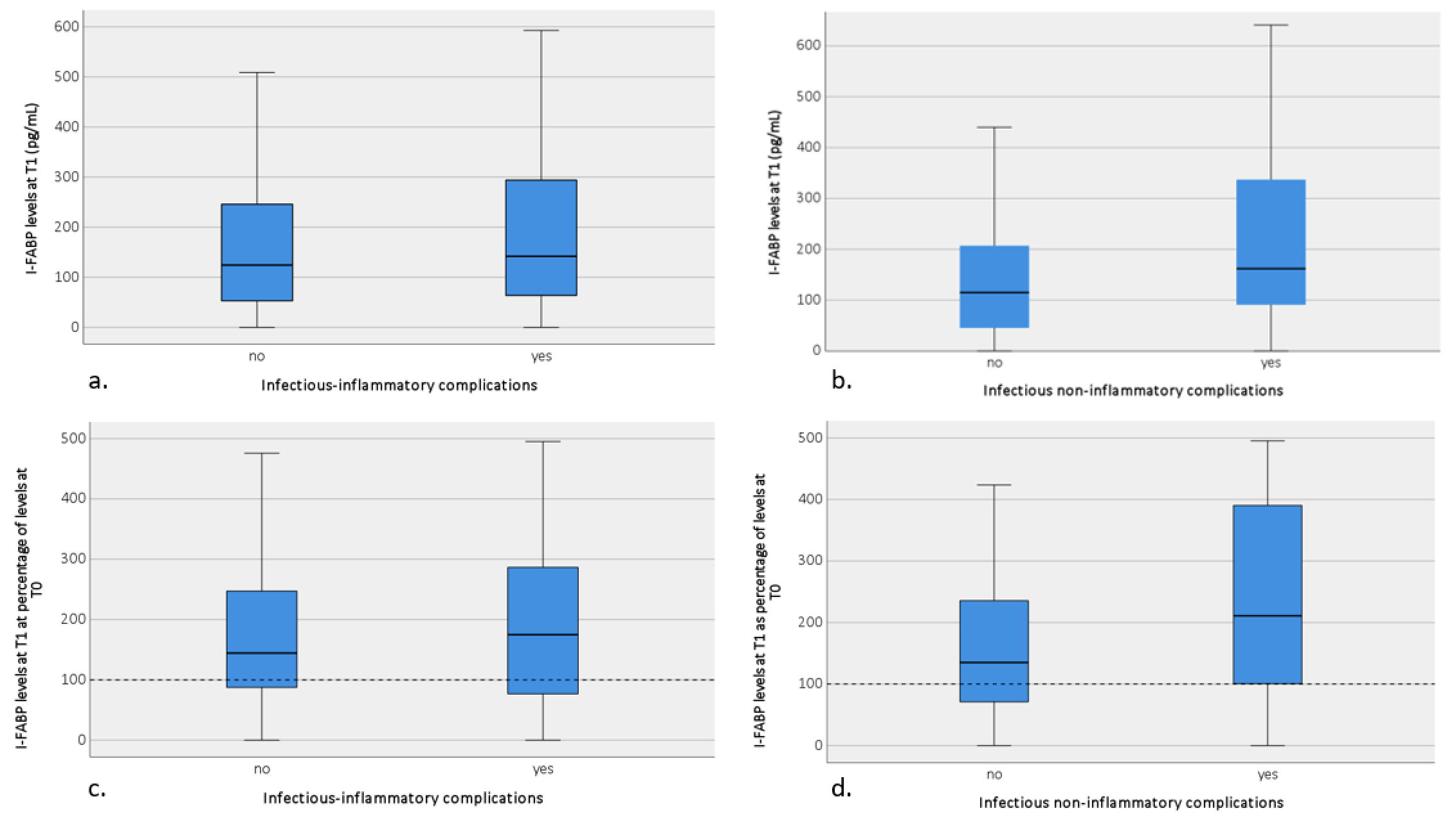


**Supplementary figure 1: I-FABP values for both patients experiencing infectious-inflammatory complications and infectious non-inflammatory complications vs patients without these complications.** a. and b. Absolute I-FAPB levels at T1. c. and d. T1 I-FABP levels as percentage of T0.
