# Supplementary material for: The Association Between Intraoperative Compromised Intestinal Integrity and Postoperative Complications in Cancer Patients
Source: Ann Surg Oncol. 2024 Jan 15;31(4):2699–708. doi: 10.1245/s10434-023-14857-7 (PMC10908644; doi:10.1245/s10434-023-14857-7)
Supplement: Supplementary file 2 — Supplementary file2 (DOCX 26 kb) [file 10434_2023_14857_MOESM2_ESM.docx]

**Supplementary tables**

| **Supplementary table 1: Patient- and disease related characteristics** | | | | | | |  |
| --- | --- | --- | --- | --- | --- | --- | --- |
| **Variable** | **All patients**  **(n=297)** | | **No I-FABP measurement day1&2 postop**  **(n=197)** | | **I-FABP measurement on day 1&2**  **(n=100)** | | **P-value*** |
| Sex  Female  Male | 143  154 | (47.1%)  (51.9%) | 103  94 | (52.3%)  (47.7%) | 40  60 | (40.0%)  (60.0%) | 0.05 |
| Age (years) | 70 | [66-76] | 72 | [68-78] | 65 | [58-71] | *<0.05* |
| Body Mass Index (Kg/m^2^) | 26.4 | [24.1-29.6] | 26.4 | [24.0-29.4] | 26.7 | [24.5-29.8] | 0.28 |
| CCI** score (without malignancy)  Peripheral vascular disease (including hypertension)  Diabetes  Chronic cardiac diseases (including AF)  Pulmonary diseases | 1  108  56  46  36 | [0-2]  (36.5%)  (18.9%)  (15.5%)  (12.2%) | 1  69  37  30  24 | [0-2]  (35.2%)  (18.9%)  (15.3%)  (12.2%) | 1  39  19  16  12 | [0-2]  (39.0%)  (19.0%)  (16.0%)  (12.0%) | 0.78  0.53  1.00  0.87  1.00 |
| Tumor site  Gastro-intestinal  Skin, soft tissue and lymph node  Gynecological  Hepatic, biliary and pancreatic  (Para)thyroid  Other (including mamma) | 127  72  57  19  15  7 | (42.8%)  (24.8%)  (19.2%)  (6.4%)  (5.1%)  (2.3%) | 78  51  45  12  9  2 | (39.6%)  (25.9%)  (22.8%)  (6.1%)  (4.6%)  (1.0%) | 49  21  12  7  6  5 | (49.0%)  (21.0%)  (12.0%)  (7.0%)  (6.0%)  (5.0%) |  |
| Tumor stage at inclusion  0/I  II  III  IV | 79  66  86  56 | (27.4%)  (23.0%)  (23.0%)  (19.5%) | 51  47  52  38 | (26.8%)  (25.1%)  (27.8%)  (20.3%) | 29  19  34  18 | (29.0%)  (19.0%)  (34.0%)  (18.0%) |  |

Legend: For dichotomous variables, n(%) is given. For continuous data median [1^st^-3^th^ quartile] is given when not normally distributed. *P-value for comparison between group of patients with- and without I-FABP measurement at D1 and D2. **CCI-score: Charlson Comorbidity Index

| **Supplementary table 2: Patient- and disease related characteristics for patients undergoing intra- vs extra-cavitary surgery** | | | | | | |  |
| --- | --- | --- | --- | --- | --- | --- | --- |
| **Variable** |  | | **Intracavitary surgery (n=212)** | | **Extra-cavitary surgery (n=85)** | | **P-value*** |
| Gender  Female  Male |  |  | 102  110 | (48.1%)  (51.9%) | 41  44 | (48.2%)  (51.8%) | 0.99 |
| Age (years) |  |  | 70 | [66-76] | 71 | [66-77] | 0.27 |
| Body Mass Index (Kg/m^2^) |  |  | 26.4 | [24.1-29.2] | 26.6 | [23.5-30.0] | 0.94 |
| CCI** score (without malignancy)  Peripheral vascular disease (including hypertension) |  |  | 1  129 | [0-2]  (61.1%) | 1  59 | [0-2]  (69.4%) | 0.99  0.18 |
| Tumor site  Gastro-intestinal  Skin, soft tissue and lymph node  Gynecological  Hepatic, biliary and pancreatic  (Para)thyroid  Other (including mamma) |  |  | 129  12  47  19  5  3 | (61.1%)  (5.7%)  (22.2%)  (9.0%)  (2.4%)  (1.4%) | 1  60  10  0  10  4 | (1.2%)  (70.6%)  (11.8%)  (0.0%)  (11.8%)  (4.7%) | <0.01 |
| Tumor stage at inclusion  0/I  II  III  IV |  |  | 56  42  57  50 | (27.3%)  (20.5%)  (27.8%)  (24.4%) | 23  24  29  6 | (28.0%)  (29.3%)  (35.4%)  (7.4%) | 0.02 |

Legend: For dichotomous variables, n(%) is given. For continuous data median [1^st^-3^th^ quartile] is given when not normally distributed. *P-value for comparison between group of patients with- and without I-FABP measurement at D1 and D2. **CCI-score: Charlson Comorbidity Index
